# An in silico approach to identify and prioritize miRNAs target sites polymorphisms in colorectal cancer and obesity

**DOI:** 10.1002/cam4.3546

**Published:** 2020-10-18

**Authors:** Morteza Gholami, Marzieh Zoughi, Bagher Larijani, Mahsa M. Amoli, Milad Bastami

**Affiliations:** ^1^ Obesity and Eating Habits Research Center, Endocrinology and Metabolism Clinical Sciences Institute Tehran University of Medical Sciences Tehran Iran; ^2^ Endocrinology and Metabolism Research Center, Endocrinology and Metabolism Clinical Sciences Institute Tehran University of Medical Sciences Tehran Iran; ^3^ Metabolic Disorders Research Center, Endocrinology and Metabolism Molecular‐Cellular Sciences Institute Tehran University of Medical Sciences Tehran Iran; ^4^ Department of Medical Genetics Faculty of Medicine Tabriz University of Medical Sciences Tabriz Iran

**Keywords:** bioinformatics, biomarkers, colorectal cancer, polymorphisms

## Abstract

Colorectal cancer (CRC) and obesity are linked clinical entities with a series of complex processes being engaged in their development. MicroRNAs (miRNAs) participate in these processes through regulating CRC and obesity‐related genes. This study aimed to develop an in silico approach to systematically identify and prioritize miRNAs target sites polymorphisms in obesity and CRC. Data from genome‐wide association studies (GWASs) were used to retrieve CRC and obesity‐associated variants. The polymorphisms that were resided in experimentally verified or computationally predicted miRNA target sites were retrieved and prioritized using a range of bioinformatics analyses. We found 6284 CRC and 38931 obesity unique variants. For CRC 33 haplotypes variants in 134 interactions were in miRNA targetome, while for obesity we found more than 935 unique interactions. Functionally prioritized SNPs revealed that, SNPs in 153 obesity and 50 CRC unique interactions were have disruptive effects on miRNA:mRNA integration by changing on target RNA secondary structure. Structural accessibility of target sites were decreased in 418 and 103 unique interactions and increased in 516 and 79 interactions, for obesity and CRC, respectively. The miRNA:mRNA hybrid stability was increased in 127 and 17 unique interactions and decreased in 33 and 24 interactions for the effect of obesity and CRC SNPs, respectively. In this study, seven SNPs with 15 interactions and three SNPs with four interactions were prioritized for obesity and CRC, respectively. These SNPs could be used for future studies for finding potential biomarkers for diagnoses, prognosis, or treatment of CRC and obesity.

## Graphical abstract

This study aimed to develop an in silico approach to systematically identify and prioritize miRNAs target sites polymorphisms in colorectal cancer and obesity. In this study, seven SNPs with 15 interactions and three SNPs with four interactions were prioritized for obesity and CRC, respectively. These SNPs could be used for future studies for finding potential biomarkers for diagnoses, prognosis, or treatment of CRC and obesity.

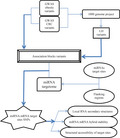

## 1 INTRODUCTION

The previous studies have witnessed growing concerns about two related clinical entities: cancer, as the second cause of death, and obesity, as a chronic inflammatory disease in the recent years. According to a report by the world health organization (WHO), obesity effects more than 1/8 of adults worldwide.^1^ A growing body of research provided evidence that functionally links obesity to the risk of several cancers including CRC.^2^, ^3^, ^4^, ^5^ A range of genetic and environmental factors contributes to the risk of CRC and obesity. Experimentations have documented a range of pathological molecular alterations contributing to CRC and obesity, among which dysregulation of miRNAs is specially highlighted.

MiRNAs are small noncoding RNAs that regulate tumorigenesis through functioning as either tumor suppressors or oncogenes.^6^ They participate in transcriptome regulation by binding to 3'‐untranslated region (3'‐UTR) of target mRNAs and either suppressing translation or inducing mRNA degradation. It has been shown that the intricate miRNA:mRNA network may be influence by the presence of single nucleotide polymorphism (SNP) within or near miRNA binding site. Such miRNA binding site polymorphism may influence miRNA:mRNA interaction through altering miRNA:mRNA hybrid stability, secondary structure of local RNA, structural accessibility of target sites, or even creating novel biding sites. Several recent genetic association studies have highlighted the contribution of miRNA binding site polymorphisms to the risk of complex disease specially CRC.^7^, ^8^ In this study, we leveraged data from genome‐wide association studies (GWASs) on CRC, obesity, and obesity‐related traits to explore putative disease‐associated polymorphisms that influence miRNA target sites. A range of bioinformatics analyses was also performed to predict functional consequences of these polymorphisms and provide a list of prioritized variants for future experimentations.

## 2 METHOD AND ANALYSIS

**FIGURE 1:**
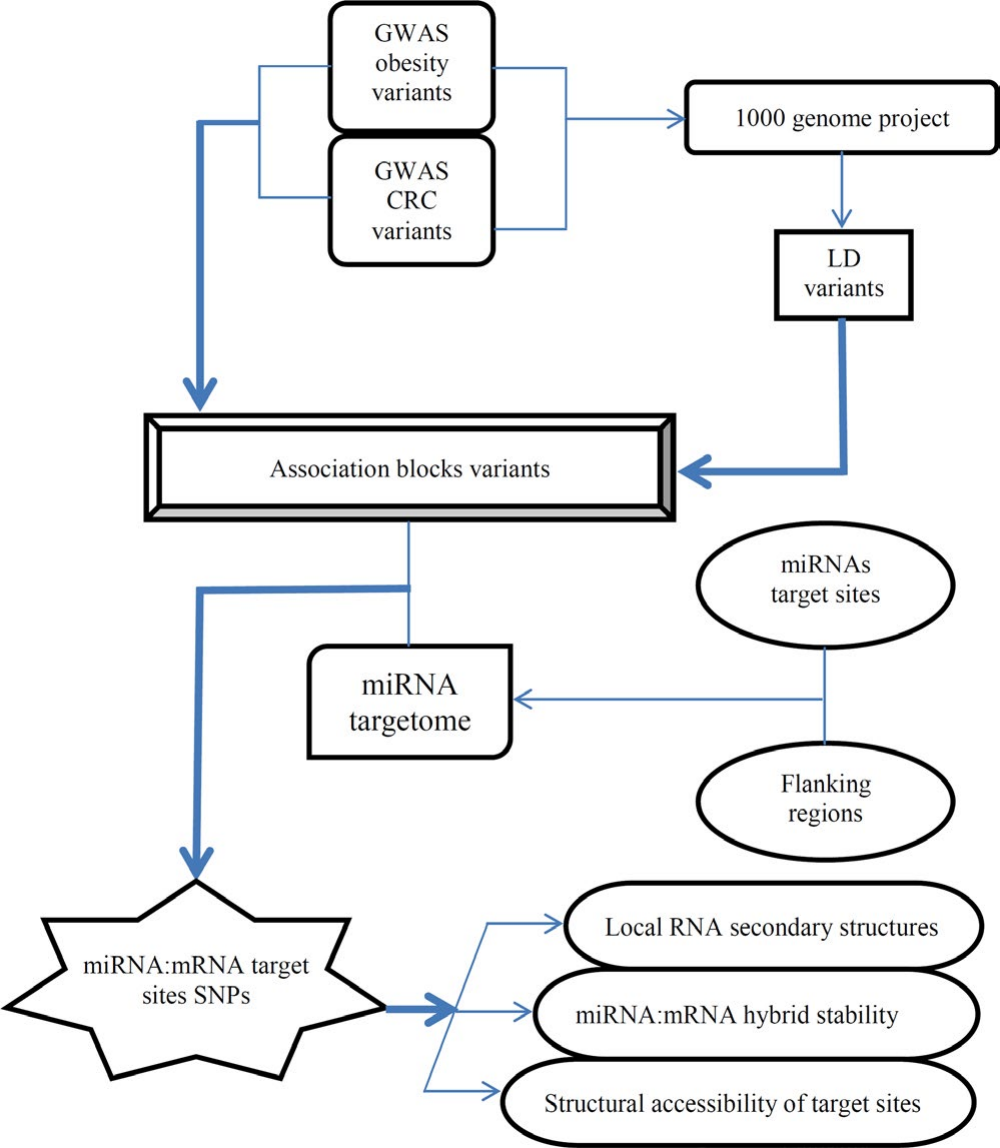
The study's workflow. See method for details

The bioinformatics methods applied in this study are depicted in Figure 1. Variants from GWA studies on CRC risk, CRC survival, obesity risk, and obesity‐related traits were retrieved from NHGRI‐EBI GWAS catalog (gwas_catalog_v1.0.1‐associations_e90_r2017‐10‐10), available at (https://www.ebi.ac.uk/gwas/). The HaploReg (version 4.1) database was used to construct population‐specific association blocks for each GWAS lead variant based on 1000 Genome project Phase I populations and a defined linkage disequilibrium threshold (i.e., *r* ^2^ of at least 0.6). The obtained association blocks, containing all putative disease‐associated variants were intersected with a miRNA targetome data set to identify putative disease‐associated variants that are resided within or in flanks of an experimentally verified or a computationally predicted miRNAs target site. The functional effects of these polymorphism on different aspects of miRNA:mRNA interactions, including local RNA secondary structures, structural accessibility of target sites, and miRNA:mRNA hybrid stability, were analyzed.

### 2.1 The MIRNA targetome data set

A comprehensive data set of experimentally verified and computationally predicted miRNA target sites were obtained by combining miRNA:mRNA interactions from the StarBase (version 2, available at http://starbase.sysu.edu.cn/starbase2/index.php), the targetScan (version 7.1, available at http://www.targetscan.org/vert_71/), and the microRNA.org (available at http://www.microrna.org/microrna/home.do) databases. The data set includes both computationally predicted target sites and those that are validated using a range of methods such as CLIP‐Seq.^9^ The 25 nucleotides upstream and downstream of target sites were considered as flanking regions.

### 2.2 Putative CRC/obesity‐associated variants and association blocks

The lead SNP of GWA studies with a *p*‐value ≤1.0 × 10^−6^ were retrieved. The HaploReg (version 4.1, available at https://pubs.broadinstitute.org/mammals/haploreg/haploreg.php) was used to retrieve all proxy SNPs (defined as *r* ^2^≥0.6) of GWAS lead variants based on 1000 Genomes project super‐populations.^10^ Five categories of obesity‐related traits were considered (Table 1). Moreover, we obtained GWAS index variants from GWA studies associated with CRC risk and CRC survival.

**TABLE 1:** Categories of obesity‐associated traits obtained from GWAS catalog

Abbreviation	Categories	Covered traits
Ob	Obesity	Adiposity, Obesity, Overweight, Extreme obesity, Early onset extreme obesity
W‐B	Weight & BMI	body mass index (BMI), body weight, Childhood BMI, Weight z‐score, Birth weight
WHR	Waist/hip circumference(ratio)	Waist circumference, Hip circumference, Waist‐hip ratio
AT	Adipose tissue	Visceral adipose tissue (VAT), Subcutaneous adipose tissue (SAT), AT/SAT ratio, Visceral fat
FM	Fat mass	Body fat mass, Trunk fat mass, Body fat percentage, newborns Fat mass

### 2.3 Effect of targetome SNPS on local RNA secondary structures

The RNAsnp program (version 1.2, available athttps://rth.dk/resources/rnasnp/)^11^ used to assess impacts of CRC or obesity‐associated SNPs in target sites and flanking regions on secondary structures of local RNA. RNA sequences and SNPs were inputted in RNAsnp to generate probability matrix associated to wild‐type (WT) and mutant (ALT) alleles. RNAsnp shows the structural difference between WT and ALT alleles with the Euclidean distance measure (*d*) for all sequence intervals and reports the polymorphism with the maximum base pair distances (*d*_max_) and the corresponding p‐value. A p‐value less than 0.2 is considered significant.^11^

### 2.4 Structural accessibility of target sites

As described previously, changes in structural accessibility of target sites may interfere with miRNA binding. RNAplfold program was used to calculate structural accessibility for 3′‐UTRs with the ALT and WT alleles. The difference between the target site accessibility of WT and ALT allele was computed using ∆Pu.^12^, ^13^

### 2.5 MIRNA:mRNA hybrid stability

A target site polymorphism may also interfere with miRNA binding through altering the stability miRNA:mRNA hybrid structure. The RNA hybrid v2.1.2 (available at https://bibiserv.cebitec.uni‐bielefeld.de/rnahybrid/)^14^ was employed to identify free energy of hybridization (ΔGhybrid) for ALT and WT alleles. ΔΔGhybrid for each miRNA target site polymorphism (calculated as ΔΔGhybrid = ΔGhybrid assassinate to ALT–ΔGhybrid assassinate to WT), is a measure of the SNP effect on the stability of hybridization. A positive ΔΔGhybrid is an indication of a decreased stability that is imposed by the ALT allele.

## 3 RESULTS

### 3.1 Putative obesity/CRC‐associated SNPs in miRNAs targetome

We obtained 159 GWAS index SNPs from 32 studies on CRC risk and 45 GWAS index SNPs from three studies on CRC survival by mining GWAS catalog. After extending polymorphisms to association haplotypes, 5163 and 1121 putative disease variants were retrieved for CRC risk and survival, respectively. The CRC‐associated index and haplotypes variants were reported in a range of populations including Europeans (n = 4464), Asians (n = 2216), Africans (n = 53), and Americans (n = 130), with some variants being reported in multiple populations. After mapping putative CRC‐associated variants to the miRNA targetome data set, 134 and 48 unique miRNA:mRNA:SNP were identified for CRC risk and survival, respectively. In other words, 33 the CRC‐associated index and haplotypes variants were identified to reside within or near miRNA binding sites, potentially influencing 134 miRNA:mRNA:SNP interactions (Table 2). Moreover, 11 unique CRC survival‐associated variants were found to reside within or near miRNA binding sites, influencing 48 miRNA:mRNA:SNP interactions (Table 3).

**TABLE 2:** CRC risk‐related haplotype variants of GWA studies raised on miRNAs targetome

Databases	Targetome SNP	miRNA	Gene	GWAS SNP	*r* ^2^ ^a^	Population
StarBase	rs10318 C > T	miR−124‐3p	GREM1	rs2293582 G > A, rs73376930 A > C	0.79, 0.82	EUR, EUR
Microrna.org	rs10318 C > T	miR−96, miR−182, miR−1271	GREM1	rs2293582 G > A, rs73376930 A > C	0.79, 0.82	EUR, EUR
Microrna.org	rs1046097 A > G	miR−382	TUBG2	rs9901225 C > G	0.85	EUR
StarBase	rs1048165 A > C	miR−142‐3p, miR−155‐5p, miR−185‐5p	SNRNP27	rs4853036 G > A	0.7	EUR
Microrna.org	rs1048165 A > C	miR−185, miR−155	SNRNP27	rs4853036 G > A	0.7	EUR
StarBase	rs1051473 T > C	miR−433‐3p, miR−203a	LAMC1	rs10752881 A > G, rs10752881 A > G	0.7, 0.77	EUR, ASN
Microrna.org	rs1051473 T > C	miR−203	LAMC1	rs10752881 A > G, rs10752881 A > G	0.7, 0.77	EUR, ASN
Microrna.org	rs1062044 A > G	miR−96	LAMC1	rs10752881 A > G, rs10752881 A > G	0.7, 0.77	EUR, ASN
StarBase	rs11064467 C > T	miR−7‐5p, miR−328‐3p, miR−330‐3p, miR−128‐3p, miR−335‐5p	ENO2	rs11064437 C > T	0.6	ASN
Microrna.org	rs11064467 C > T	miR−7, miR−128, miR−328, miR−335	ENO2	rs11064437 C > T	0.6	ASN
Microrna.org	rs11085537 G > C	miR−140‐5p, miR−125a−3p, miR−136	LOC440518	rs11671104 A > C	0.6	EUR
Microrna.org	rs11571475 A > G	miR−17, miR−20a, miR−93, miR−106a/b, miR−20b, miR−519d	RAD52	rs12309274 T > C, rs12309274 T > C	0.82, 0.71	EUR, ASN
StarBase	rs1547715 A > G	miR−511‐5p, miR−16‐5p, miR−15a−5p, miR−431‐5p, miR−195‐5p, miR−423‐3p, miR−150‐5p, miR−15b−5p, miR−424‐5p	LAMC1	rs10752881 A > G, rs10752881 A > G	0.7, 0.77	EUR, ASN
Microrna.org	rs1547715 A > G	miR−362‐3p	LAMC1	rs10752881 A > G, rs10752881 A > G	0.7, 0.77	EUR, ASN
StarBase	rs174545 C > A	miR−452‐5p	FADS1	rs174537 G > C, rs174537 G > C	0.98, 1	EUR, ASN
StarBase	rs174546 C > T	miR−496	FADS1	rs174537 G > C, rs174537 G > C	0.98, 1	EUR, ASN
Microrna.org	rs174546 C > T	miR−496	FADS1	rs174537 G > C, rs174537 G > C	0.98, 1	EUR, ASN
Microrna.org	rs2715761 C > T	miR−203	MORC1	rs2593957 T > C	0.74	EUR
StarBase	rs295458 C > A	miR−128‐3p, miR−96‐5p	KLHL18	rs8180040 T > A	0.98	EUR
TargetScan	rs295458 C > A	miR−128‐3p, miR−1193, miR−493‐3p	KLHL18	rs8180040 T > A	0.98	EUR
Microrna.org	rs3087967 T > A	miR−9, miR−186, miR−362‐3p, miR−329	C11orf53	rs3802842 C > A, rs3802842 C > A	0.94, 0.98	EUR, ASN
Microrna.org	rs3088140 T > A	miR−494	UTP23	rs6469656 G > A, rs6469656 G > A	0.78, 0.74	EUR, ASN
StarBase	rs3184122 A > G	miR−21‐5p, miR−590‐5p	LIMA1	rs34245511 G > C	0.87	EUR
Microrna.org	rs3184122 A > G	miR−21, miR−590‐5p	LIMA1	rs34245511 G > C	0.87	EUR
StarBase	rs3359 G > C	miR−29c−3p, miR−29b−3p, miR−129‐5p, miR−16‐5p, miR−15a−5p, miR−422a, miR−195‐5p, miR−497‐5p, miR−122‐5p, miR−15b−5p, miR−424‐5p	LAMC1	rs10752881 A > G, rs10752881 A > G	0.7, 0.77	EUR, ASN
TargetScan	rs3359 G > C	miR−15‐5p, miR−16‐5p, miR−195‐5p, miR−424‐5p, miR−497‐5p	LAMC1	rs10752881 A > G, rs10752881 A > G	0.7, 0.77	EUR, ASN
Microrna.org	rs3359 G > C	miR−15a, miR−16, miR−15b, miR−195, miR−422a, miR−424, miR−497	LAMC1	rs10752881 A > G, rs10752881 A > G	0.7, 0.77	EUR, ASN
Microrna.org	rs3741698 C > A	miR−488	TBX3	rs59336 T > A	0.63	ASN
Microrna.org	rs5445 C > T	miR−183, miR−124, miR−150, miR−506	GNB3	rs11064437 C > T	0.6	ASN
StarBase	rs58194764 T > C	miR−300	TPI1	rs11064437 C > T	0.88	ASN
Microrna.org	rs58194764 T > C	miR−300	TPI1	rs11064437 C > T	0.88	ASN
StarBase	rs6424890 A > G	miR−320a/b/c/d	LAMC1	rs10752881 A > G, rs10752881 A > G	0.7, 0.77	EUR, ASN
TargetScan	rs6424890 A > G	miR−140‐5p	LAMC1	rs10752881 A > G, rs10752881 A > G	0.7, 0.77	EUR, ASN
Microrna.org	rs6424890 A > G	miR−140‐5p, miR−320a, miR−590‐3p, miR−320b, miR−320c	LAMC1	rs10752881 A > G, rs10752881 A > G	0.7, 0.77	EUR, ASN
StarBase	rs679 C > A	miR−218‐5p	MLX	rs9901225 C > G	0.72	EUR
Microrna.org	rs679 C > A	miR−103, miR−107	MLX	rs9901225 C > G	0.72	EUR
Microrna.org	rs709206 T > A	miR−137, miR−371‐5p	CABLES2	rs6061231 C > A	0.95	ASN
StarBase	rs7473 G > A	miR−33b−5p, miR−153‐3p, miR−124‐3p, miR−33a−5p, miR−448, miR−506‐3p	LAMC1	rs10752881 A > G, rs10752881 A > G	0.7, 0.77	EUR, ASN
TargetScan	rs7473 G > A	miR−124‐3p.1, miR−124‐3p.2, miR−506‐3p, miR−33‐5p	LAMC1	rs10752881 A > G, rs10752881 A > G	0.7, 0.77	EUR, ASN
Microrna.org	rs747949 G > A	miR−218	CABLES2	rs6061231 C > A	0.95	ASN
StarBase	rs8079855 A > C	let−7c−5p	FAM134C	rs9901225 C > G	0.72	EUR
StarBase	rs8668 A > G	miR−342‐3p, miR−216a−5p, miR−155‐5p	CABLES2	rs2427308 C > T, rs6061231 C > A	0.7, 0.91	EUR, ASN
Microrna.org	rs8668 A > G	miR−216a, miR−218, miR−155, miR−342‐3p	CABLES2	rs2427308 C > T, rs6061231 C > A	0.7, 0.91	EUR, ASN
StarBase	rs8853 T > C	miR−203a, miR−211‐5p, miR−204‐5p,	TBX3	rs59336 T > A, rs59336 T > A	0.82, 0.94	EUR, ASN
Microrna.org	rs8853 T > C	miR−204, miR−211	TBX3	rs59336 T > A, rs59336 T > A	0.82, 0.94	EUR, ASN
TargetScan	rs888208 A > G	miR−205‐5p	NKX2‐3	rs12412391 A > G, rs12412391 A > G	0.99, 0.95	EUR, ASN
Microrna.org	rs888208 A > G	miR−205	NKX2‐3	rs12412391 A > G, rs12412391 A > G	0.99, 0.95	EUR, ASN
StarBase	rs9364 G > A	miR−346	LIMA1	rs34245511 G > C	0.92	EUR
Microrna.org	rs9364 G > A	miR−194	LIMA1	rs34245511 G > C	0.92	EUR
Microrna.org	rs9375 C > T	miR−141, miR−142‐3p, miR−9, miR−200a, miR−494, miR−495, miR−539	RBM16	rs7740797 G > C	0.85	EUR
StarBase	rs944970 T > C	miR−216a−5p	LAMC1	rs10752881 A > G, rs10752881 A > G	0.7, 0.77	EUR, ASN
Microrna.org	rs944970 T > C	miR−25, miR−92a, miR−216a, miR−367	LAMC1	rs10752881 A > G, rs10752881 A > G	0.7, 0.77	EUR, ASN
StarBase	rs944971 T > C	miR−24‐3p, miR−506‐3p	LAMC1	rs10752881 A > G, rs10752881 A > G	0.7, 0.77	EUR, ASN
TargetScan	rs944971 T > C	miR−24‐3p	LAMC1	rs10752881 A > G, rs10752881 A > G	0.7, 0.77	EUR, ASN
Microrna.org	rs944971 T > C	miR−24	LAMC1	rs10752881 A > G, rs10752881 A > G	0.7, 0.77	EUR, ASN

Abbreviations: AMR, American; ASN, Asian; EUR, European.

^a^ *r* ^2^ (squared Pearson's correlation). SNPs in interactions related to under lined miRNAs located in miRNA:miRNA:SNP target site, others located in flanking region in targetome.

**TABLE 3:** CRC survival‐associated variants raised on miRNAs targetome

Databases	Targetome SNP	miRNA	Gene	GWAS SNP	*r* ^2^ ^a^	Population
Microrna.org	rs1127466 G > C	miR−338‐3p	SLC22A23	rs4959799 G > C	0.78	EUR
StarBase	rs1127470 T > A	miR−302e, miR−183‐5p	SLC22A23	rs4959799 G > C	0.75	EUR
Microrna.org	rs2076472 T > C	miR−431	APOBEC2	rs2073016 T > C	1	EUR
StarBase, Microrna.org	rs4229 A > C	miR−125a−3p	LARP4B	rs1555895 A > C	0.62	EUR
StarBase	rs45629235 A > G	miR−432‐3p, miR−27a−3p, miR−27b−3p	SLC22A23	rs4959799 G > C	0.78	EUR
StarBase	rs7703 C > G	miR−124‐3p	LARP4B	rs1555895 A > C	0.69	EUR
Microrna.org	rs9501973 T > C	miR−203	SLC22A23	rs4959799 G > C	0.78	EUR
StarBase	rs9501974 T > C	miR−181b−5p, miR−1297, miR−181c−5p, miR−182‐5p, miR−96‐5p	SLC22A23	rs4959799 G > C	0.78	EUR
Microrna.org	rs9501974 T > C	miR−181b, miR−181c	SLC22A23	rs4959799 G > C	0.78	EUR
StarBase	rs9501975 C > A	miR−181b−5p, miR−1297, miR−17‐5p, miR−20a−5p, miR−203a, miR−181c−5p, miR−106b−5p, miR−182‐5p, miR−96‐5p, miR−20b−5p, miR−106a−5p	SLC22A23	rs4959799 G > C	0.78	EUR
Microrna.org	rs9501975 C > A	miR−181b, miR−181c	SLC22A23	rs4959799 G > C	0.78	EUR
StarBase	rs9503516 G > A	miR−137,miR−9‐5p, let−7a−5p, let−7i−5p, miR−1297, miR−4500, let−7e−5p, let−7c−5p, let−7b−5p, let−7 g−5p, miR−4458, let−7f−5p, let−7d−5p, miR−98‐5p	SLC22A23	rs4959799 G > C	0.78	EUR
TargetScan	rs9503516 G > A	miR−9‐5p, miR−137	SLC22A23	rs4959799 G > C	0.78	EUR
Microrna.org	rs9503516 G > A	miR−137	SLC22A23	rs4959799 G > C	0.78	EUR
StarBase	rs9503517 C > T	miR−137, miR−1297	C6orf85	rs4959799 G > C	0.78	EUR
Microrna.org	rs9503517 C > T	miR−137	SLC22A23	rs4959799 G > C	0.78	EUR

Abbreviation: EUR, European.

^a^ *r* ^2^(squared Pearson's correlation).SNPs in interactions related to under lined miRNAs located in miRNA:miRNA:SNP target site, others located in flanking region in targetome.

We retrieved 1079 GWAS index variants from 75 studies pertaining to five obesity‐related categories. After extending to association blocks, 38931 unique putative obesity‐related variants were obtained. Since the most of included GWA studies were related to European populations, the more of putative obesity‐related variants belonging to the European. Table 4 summarized number of variants in each super‐population. Variants from our results were intersected which targetome intervals. Finally, we find 935 miRNA:mRNA:SNP unique interactions, in which 198 of them were in miRNAs binding sites and others were in its up or down stream. In other words, 196 putative obesity‐associated variants were identified to reside within or near miRNA binding sites. All interactions, GWAS SNPs, and populations are shown in supplementary file 1. Seventeen and 125 unique miRNA:mRNA:SNP interactions (seven and 16 unique SNPs) were find for GWAS obesity‐related lead variants in miRNAs binding sites and its flanking regions, respectively, which are presented in Tables 5 and 6.

**TABLE 4:** Number of obesity‐related variants included in this study based on 1000 genomes project super‐populations

Categories	Population	Index variants	Proxy variants	Combined
Ob	EUR	123	6074	6197
	ASN	12	610	622
W‐B	EUR	534	28323	28857
	ASN	417	20378	20795
	AFR	396	7099	7495
	AMR	334	13071	13405
WHR	EUR	163	7106	7269
	ASN	155	6762	6917
	AFR	149	1991	2140
	AMR	29	384	413
AT	EUR	138	3758	3896
	AFR	6	146	152
FM	EUR	26	1595	1621
	ASN	15	1046	1061
	AFR	15	491	506
	AMR	50	1841	1891
Overall	All	2562	100675	103237
Overall without duplicates^a^	All	1079	37852	38931

Abbreviations: AFR, African; AMR, American; ASN, Asian; AT, Adipose tissue; EUR, European; FM, Fat mass; Ob, Obesity; W‐B, Weight & BMI; WHR, Waist/hip circumference (ratio).

^a^ Number of unique index and proxy variants in five obesity‐related categories.

**TABLE 5:** Obesity‐related GWAS lead variants raised on miRNAs target sites

Categories	SNPs	Gene	miRNA(s)	Population(s)	Database
W‐B	rs4395360 C > T	PQLC2L	miR−205	EUR, ASN, AMR, AFR	Microrna.org
W‐B	rs715 T > A	CPS1	miR−432‐5p	EUR, ASN, AMR, AFR	StarBase
WHR	rs2179129 A > G	ZNRF3	miR−411‐5p	EUR, ASN, AFR	StarBase, TargetScan
WHR	rs2179129 A > G	ZNRF3	miR−379, miR−411,	EUR, ASN, AFR	Microrna.org
AT	rs1048497 G > A	ZNF664	miR−17‐3p, miR−487b−3p, miR−124‐3p	EUR	StarBase
AT	rs1048497 G > A	ZNF664	miR−139‐5p, miR−487b	EUR	Microrna.org
Ob, W‐B	rs7132908 G > A	FAIM2	miR−326	EUR, EUR, AMR	TargetScan
Ob, W‐B	rs9299 C > A	HOXB‐AS3	miR−222‐3p	EUR, EUR	StarBase
Ob, W‐B	rs9299 C > A	HOXB5	miR−7	EUR, EUR	Microrna.org
Ob, FM	rs6857 C > T	NECTIN2	miR−339‐5p	EUR, ASN, AMR, AFR	StarBase, Microrna.org

Abbreviations: AFR, African; AMR, American; ASN, Asian; AT, Adipose tissue; EUR, European; Ob, Obesity; FM, Fat mass; W‐B, Weight & BMI; WHR, Waist/hip circumference (ratio).

**TABLE 6:** Obesity‐related GWAS lead variants raised on flanking regions of miRNA target sites

Categories	SNPs	Gene	miRNA(s)	Population(s)	Database	
W‐B	rs715 T > A	CPS1	miR−190b	EUR, ASN, AMR, AFR		StarBase, Microrna.org
Ob	rs1048466 G > A	CCDC77	miR−184	EUR	StarBase, Microrna.org	
Ob	rs1048466 G > A	CCDC77	miR−542‐3p	EUR	Microrna.org	
AT	rs1056053 C > A	TBXT	miR−106a, miR−17, miR−218	EUR	Microrna.org	
AT	rs1048497 G > A	ZNF664	miR−758	EUR	Microrna.org	
AT	rs1048497 G > A	ZNF664	miR−758‐3p	EUR	StarBase	
Ob, W‐B	rs9299 C > A	HOXB5	miR−185	EUR	Microrna.org	
Ob, W‐B	rs9299 C > A	HOXB5	miR−185‐5p, miR−7‐5p	EUR	StarBase	
Ob, W‐B	rs7132908 G > A	FAIM2	miR−185	EUR	Microrna.org	
Ob, W‐B, WHR	rs2531995 C > T	ADCY9	miR−193a−3p	EUR, ASN, AFR	StarBase	
Ob, W‐B, WHR	rs2531995 C > T	ADCY9	miR−24‐3p	EUR, ASN, AFR	StarBase, TargetScan	
Ob, W‐B, WHR	rs879620 C > T	ADCY9	miR−506‐3p	EUR, ASN, AFR	StarBase	
W‐B	rs10540 G > A	RNH1	miR−15a−5p, miR−15b−5p, miR−16‐5p	EUR, ASN, AMR, AFR	StarBase, Microrna.org	
W‐B	rs715 T > A	CPS1	miR−190a−5p	EUR, ASN, AMR, AFR	StarBase, Microrna.org	
W‐B	rs10540 G > A	RNH1	miR−195‐5p	EUR, ASN, AMR, AFR	StarBase, Microrna.org	
W‐B	rs4395360 C > T	C3orf55	miR−199a−5p, miR−199b−5p, miR−300, miR−381	EUR, ASN, AMR, AFR	Microrna.org	
W‐B	rs10540 G > A	RNH1	miR−424‐5p	EUR, ASN, AMR, AFR	StarBase, Microrna.org	
W‐B	rs715 T > A	CPS1	miR−450a	EUR, ASN, AMR, AFR	Microrna.org	
W‐B	rs715 T > A	CPS1	miR−496, miR−450a−5p	EUR, ASN, AMR, AFR	StarBase, Microrna.org	
W‐B	rs10540 G > A	RNH1	miR−497‐5p	EUR, ASN, AMR, AFR	StarBase, Microrna.org	
W‐B, FM	rs6857 C > T	PVRL2	miR−124	EUR, ASN, AMR, AFR	Microrna.org	
W‐B	rs6857 C > T	PVRL2	miR−506	EUR, ASN, AMR, AFR	Microrna.org	
W‐B, WHR	rs1042725 C > T	HMGA2	let−7a−5p	EUR	StarBase, TargetScan, Microrna.org	
W‐B, WHR	rs1042725 C > T	HMGA2	let−7b−5p, let−7c−5p, let−7d−5p, let−7e−5p, let−7f−5p, let−7 g−5p, let−7i−5p, miR−181a−5p, miR−181b−5p, miR−181c−5p, miR−181d−5p, miR−196a−5p, miR−196b−5p, miR−98‐5p	EUR	StarBase, Microrna.org	
W‐B, WHR	rs1042725 C > T	HMGA2	let−98‐5p	EUR	TargetScan	
W‐B, WHR	rs1042725 C > T	HMGA2	miR−103, miR−107, miR−15a, miR−15b, miR−16, miR−181a	EUR	Microrna.org	
W‐B, WHR	rs1042725 C > T	HMGA2	miR−196‐5p	EUR	TargetScan, Microrna.org	
W‐B, WHR	rs1042725 C > T	HMGA2	miR−202‐3p, miR−445, miR−4500	EUR	StarBase	
W‐B, WHR	rs1042725 C > T	HMGA2	miR−424, miR−497, miR−503	EUR	Microrna.org	
W‐B, WHR	rs1042725 C > T	HMGA2	miR−543	EUR	StarBase, TargetScan	
WHR	rs1045241 C > T	TNFAIP8	miR−1271‐5p, miR−182‐5p	EUR, ASN, AFR	StarBase, TargetScan, Microrna.org	
WHR	rs3087591 A > C	EVI2B	miR−190, miR−190b, miR−197, miR−590‐3p	EUR, ASN, AFR	Microrna.org	
WHR	rs1045241 C > T	TNFAIP8	miR−96‐5p	EUR, ASN, AFR	StarBase, TargetScan, Microrna.org	
WHR	rs2179129 A > G	ZNRF3	let−7a−5p, let−7f−5p, miR−130a−3p, miR−130b−3p, miR−152‐3p, miR−301a−3p, miR−301b, miR−3666, miR−379‐5p, miR−4295, miR−4500	EUR, ASN, AFR	StarBase	
WHR	rs2179129 A > G	ZNRF3	miR−130‐3p, miR−301‐3p	EUR, ASN, AFR	TargetScan	
WHR	rs2179129 A > G	ZNRF3	miR−454‐3p	EUR, ASN, AFR	StarBase, TargetScan	
FM	rs28381552 T > C	ORC3L	miR−106a−5p, miR−106b−5p, miR−122‐5p, miR−144‐3p, miR−18a−5p, miR−20a−5p, miR−302a−3p, miR−373‐3p, miR−512‐3p, miR−519a−3p, miR−519b−3p, miR−520b, miR−520c−3p, miR−520d−3p, miR−520f−3p, miR−93‐5p	AMR	StarBase	
FM	rs28381552 T > C	ORC3L	miR−144	AMR	Microrna.org	

Abbreviations: AFR, African; AMR, American; ASN,Asian; EUR, European; FM, Fat mass; Ob, Obesity; W‐B, Weight & BMI; WHR, Waist/hip circumference (ratio).

### 3.2 Effect of targetome SNPS on local RNA secondary structures

**FIGURE 2:**
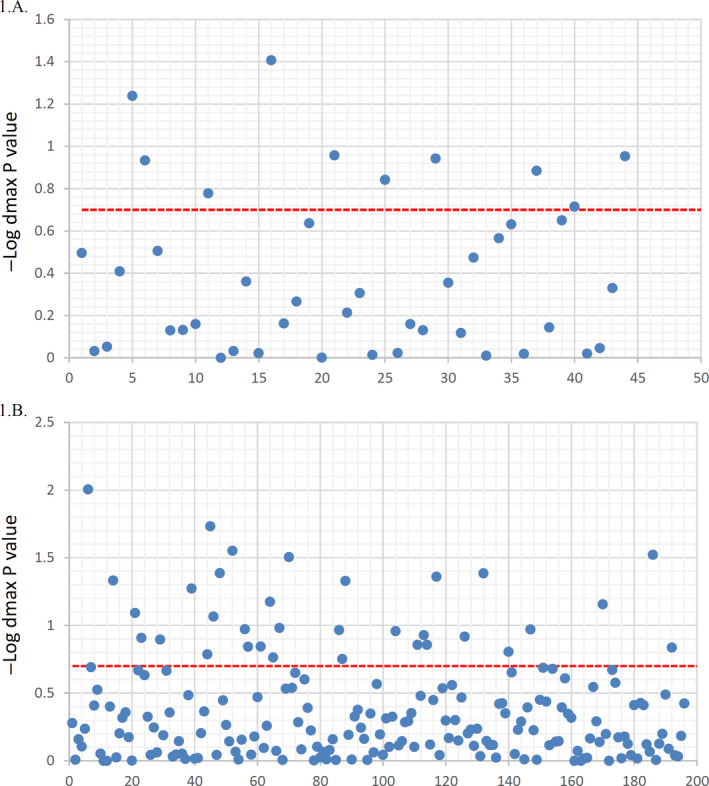
Effect of targetome SNPs on local RNA secondary structures. *d*_max_ *p*‐value distribution for CRC 1.A. and for obesity 1.B. The numbers in X‐axis are related to SNPs names and rsID (more detailed supplementary 2, sheet 3)

Previous studies showed that miRNA targetome variants can impose local structural changes^15^, ^16^, ^17^ that may not be quantified using ΔΔGhybrid. The RNAsnp outputs a p‐value that measures the significance of the observed the maximum base pair distances (*d*_max_) imposed by the variant. The analysis showed that 33 (17%) obesity related and 10 (23%) CRC‐associated variants residing within or near target site had local disruptive effects on target RNA secondary structure, which including 153 from 935 obesity related, 41 from 134 CRC risk related, and 9 from 48 CRC survival unique‐associated interactions. Data are shown in Figure 2 (*d*_max_ *p*‐value distribution of CRC and obesity) and supplementary file 2 (*d*_max_ *p*‐value for each SNPs).

### 3.3 Structural accessibility of target sites

**FIGURE 3:**
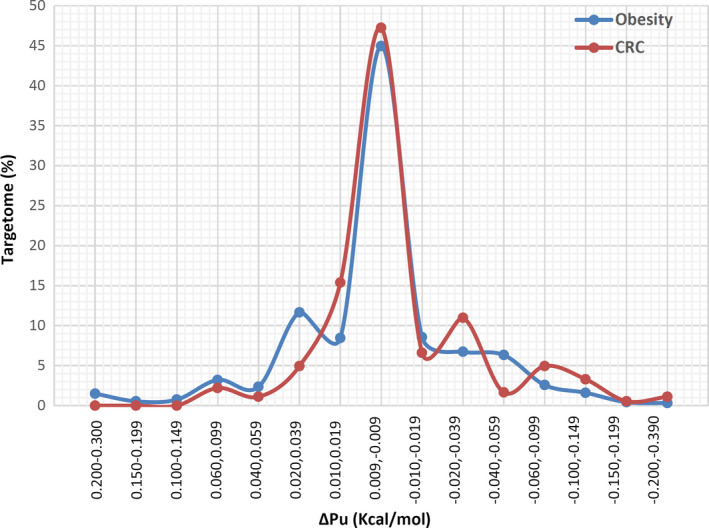
The plots show the effect of targetome SNPs on local RNA secondary structures, ΔPu for CRC and obesity shows changes in target site accessibility (Kcal/mol)

Accessibility of target site plays an important role in miRNA‐mediated regulation of gene expression.^16^, ^18^ The impact of CRC/obesity‐associated variants residing within or near miRNA binding sites on accessibility of target sites is measure by ΔPu. For obesity, we found a decreasing accessibility effect 45% (up to −37%) and increasing effect for 55% (up to 30%) of target sites, which 418 unique interactions decreased and 516 interactions increased accessibility effect. While the results for CRC were 67% (up to −39%) and 43% (form up to 9%), respectively. In another word, for CRC survival 24 unique interactions shows decreased and 24 interactions shows increased accessibility effect, and for CRC risk 79 interactions decreased while 55 interactions increased accessibility effect. These results shown that a significant numbers of target site SNPs could effect on the role of miRNA on target RNA and consequently on the role of these genes on CRC and obesity. Detailed results are shown in Figure 3 and supplementary file 3.

### 3.4 MIRNA:mRNA hybrid stability

**FIGURE 4:**
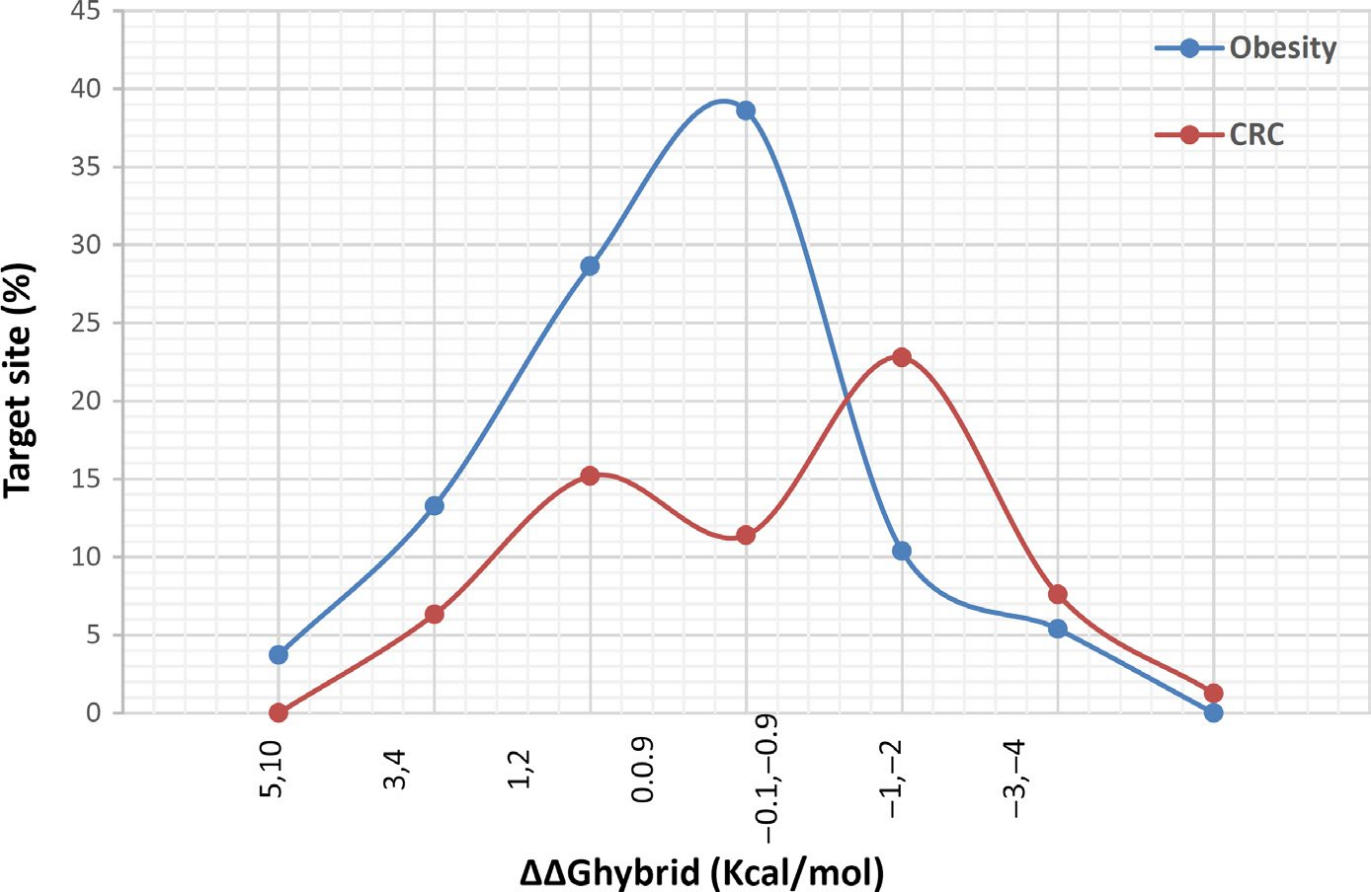
The plots show miRNA:mRNA hybrid stability based on ΔΔGhybrid of target sites

The effects on target site variant on interaction between miRNA and its target gene can be measured by change in hybrid stability. This alteration is based on base pair creation or disruption.^16^ Here, we applied ΔΔGhybrid to measure target‐SNPs effects on included miRNA–mRNA interactions. The miRNA:mRNA hybrid stability was increased in 127 interactions (up to 6.6 kcal/mol) and 17 interactions (up to 4.5 kcal/mol) for the effect of obesity and CRC SNPs, respectively, while decreased in 33 interactions (up to −2.4 kcal/mol) and 24 interactions (up to −4.7 kcal/mol) for them. The results are shown in Figure 4.

Finally, we prioritize interactions for CRC and obesity based on effect of targetome SNPs on local RNA secondary structures, structural accessibility of target sites, miRNA:mRNA hybrid stability, and annotation of polymorphisms with expression quantitative trait loci (eQTL). To validate the prioritized genes, as well as to investigate the association between the obesity genes with CRC, we analyzed the genes in TCGA data (available at https://portal.gdc.cancer.gov/).^19^ The obtained results are presented in Table 7.

**TABLE 7:** Prioritized SNPs in target site associated to obesity or CRC

Databases	Categories	SNP	Reference‐GWAS SNP	Is SNP eQTL?	GERP^a^	SiPhy^a^	RNA secondary structures		Structural accessibility	miRNA:mRNA hybrid stability	Interaction	Gene survival effect (according to TCGA data)	
							d_max	*p*‐value	ΔPu%	ΔΔGhybrid		*p*‐value^b^	*p*‐value^c^
StarBase, Microrna.org	W‐B	rs16912239A>C	rs16912238A>G	No	+	+	0.3988	0.01	2.76	3.9	hsa‐miR−30a−5p:PDCL:rs16912239	0.099	0.186
									2.59	3.1	hsa‐miR−30c−5p:PDCL:rs16912239		
									2.76	3.9	hsa‐miR−30d−5p:PDCL:rs16912239		
									2.47	3.9	hsa‐miR−30b−5p:PDCL:rs16912239		
									2.76	3.9	hsa‐miR−30e−5p:PDCL:rs16912239		
StarBase, Microrna.org	W‐B	rs1044390 T > A	rs1878931G>C	Yes	−	−	0.2875	0.03	−8.78	0.7	hsa‐miR−504‐5p:ZNF434:rs1044390	**˂0.001**	**0.001**
Microrna.org	W‐B	rs11755593G>A	rs943466G>A	Yes	−	+	0.3066	0.04	1.76	2.7	hsa‐miR−34c−5p:LEMD2:rs11755593	0.283	0.066
StarBase, Microrna.org	W‐B	rs5872A>C	rs2844479A>C	Yes	−	+	0.2219	0.07	9.96	1.7	hsa‐miR−488‐3p:CSNK2B:rs5872	0.801	0.503
Microrna.org	W‐B	rs1055816G>A	rs492400C>T	Yes	−	−	0.1652	0.10	0.20	6	hsa‐miR−15b−5p:PLCD4:rs1055816	**0.042**	0.086
									0.30	4.2	hsa‐miR−195‐5p:PLCD4:rs1055816		
									0.30	6	hsa‐miR−497‐5p:PLCD4:rs1055816		
									0.24	4.3	hsa‐miR−15a−5p:PLCD4:rs1055816		
									0.20	2.1	hsa‐miR−16‐5p:PLCD4:rs1055816		
StarBase, Microrna.org	WHR	rs4759058C>A	rs1443512A>C	Yes	−	−	0.1976	0.11	−8.47	6.6	hsa‐miR−503‐5p:HOXC13:rs4759058	**˂0.001**	**˂0.001**
StarBase, Microrna.org	WHR	rs1045475A>G	rs2531992A>G	Yes	−	+	0.1696	0.14	−1.09	0.8	hsa‐miR−141‐3p:ADCY9:rs1045475	**0.005**	**0.008**
													
StarBase	CRC risk	rs9364G>A	rs34245511G>C	Yes	−	−	0.1531	0.11	−11.19	1.9	hsa‐miR−346:LIMA1:rs9364	**0.009**	**˂0.001**
StarBase	CRC survival	rs9501974 T > C	rs4959799G>C	Yes	−	+	0.1541	0.17	1.99	1.4	hsa‐miR−182‐5p:SLC22A23:rs9501974	0.133	**0.014**
									1.99	1.4	hsa‐miR−96‐5p:SLC22A23:rs9501974		
Microrna.org	CRC risk	rs11085537G>C	rs11671104A>C	Yes	−	−	0.1129	0.19	−7.68	2.3	hsa‐miR−136:LOC440518:rs11085537	0.849	0.222

Abbreviation: eQTL, Expression quantitative trait loci.

^a^ GERP and SiPhy are measures of evolutionary conservation calculated by PhastCons algorithm.

^b^ *p*‐value related to survival rate of mutated CRC cases (PDCL n = 24, ZNF434 n = 50, LEMD2 n = 26, CSNK2B n = 31, PLCD4 n = 33, HOXC13 n = 24, ADCY9 n = 66, LIMA1 n = 37, SLC22A23 n = 38, LOC440518 n = 39) compared to unmuted (n = 606) CRC cases, form TCGA data.

^c^ *p*‐value related to survival rate of mutated CRC cases compared to overall mutated CRC case in 10 genes (n = 182).

## 4 DISCUSSION

Obesity is one of the most common complex diseases in the world and CRC is a common obesity‐related cancer. Recently many GWA studies focused on discovering the variants related to these diseases and found many novel polymorphisms. According to these data several genes appears to be involved in these diseases pathogenesis while different miRNAs have a role in their regulation. Thus, variants in miRNA:mRNA binding sites may play important role in CRC and obesity. Many of the newly identified associations in GWAS are related to noncoding regions variants.^20^ The role of these variants in tumor development and cancers risk were previously investigated in many studies.^21^, ^22^, ^23^ These variants can be prioritized by bioinformatics analyses without any cost and laboratory works which could be used in future experimental studies. Here, we used a bioinformatics approach to find polymorphisms which could potentially effect these diseases, and finally, we prioritized the most important miRNA:mRNA interactions based on using different bioinformatics tools to find the functional significance of these variants. The effect of miRNAs binding site polymorphisms on the binding sites structural changes have been studied previously^16^, ^24^ which may influence on miRNA regulatory effect. In our study, 10 and 33 miRNA binding site SNPs significantly changed on local RNA secondary structures in obesity and CRC, respectively. According to the miRNA:mRNA hybrid stability plot (Figure 4) for obesity most of SNPs in target sites do not have any effect on hybrid stability but for CRC most of miRNA binding site SNPs were effective on hybrid stability. While about the accessibility of target sites most of CRC and obesity‐related SNPs do not have any significant effect. Finally, seven SNPs with 15 interactions and three SNPs with four interactions were identified for obesity and CRC, respectively. These results revealed that miRNA:mRNA hybrid stability, structural accessibility, and RNA secondary structures could be influenced by obesity or CRC‐associated variants in miRNA target sites. We used different common algorithms for miRNA:mRNA interaction prediction with highly conserved target site to manage balance between specificity and sensitivity of bioinformatics algorithms.

According to our knowledge, all prioritized SNPs (10 SNPs in Table 7) were not studied for the association with obesity or CRC. About the association of these SNPs with obesity or CRC, only rs4759058 was predicted which to be related to waist‐hip ratio.^25^ From them rs16912239 and rs11085537 were not eQTL SNPs. We finally found that two and six miRNA binding site SNPs have related to obesity and CRC, respectively.

The TCGA data analysis validated prioritized CRC‐related genes and displayed that the obesity prioritized genes were also effective on CRC. Based on the obtained results, the ZSCAN32, PLCD4, HOXC13, and ADCY9 from obesity predicted genes, as well as LIMA1, and SLC22A23 from CRC predicted genes were significantly related to CRC survival rate. Furthermore, there are several lines of evidence on the role of prioritized genes in pathogenesis of CRC or obesity. The GWA studies confirm that, the genes in prioritized interactions for CRC were associated to the cancer.^26^, ^27^, ^28^ The expression of these genes have been considered in the recent studies, for example, the GOLGA2 newly identified as novel differentially expressed proteins in CRC,^29^ the expression of LIMA1 changed in Cholangiocarcinoma,^30^ and the expression of SLC22A23 expression increased in subjects with Laryngeal squamous cell carcinoma.^31^ Besides, the CRC prioritized genes were also associated to obesity for instance, the LIMA1,^32^ SLC22A23,^33^ and LOC440518^34^ related to body fat distribution, obesity‐related traits, and BMI, respectively.

The association of predicted obesity genes with obesity approved in the GWA studies.^33^, ^35^, ^36^, ^37^, ^38^, ^39^ For instance, LEMD2 is associated with body weight,^38^ HOXC13 and ADCY9 polymorphisms are associated with waist‐to‐hip ratio and BMI, respectively.^39^, ^40^, ^41^ The GWAS and recent published studies significantly proved the genes and miRNAs in obesity prioritized interaction were also associated to cancer. For instance, the ADCY9,^40^ and HOXC13^41^ were related to cancer, and adverse response to chemotherapy, respectively. The ADCY9 expression remarkably increased in colon tumor tissue and can be a poor prognostic factor for colon survival.^42^ The HOXC13 and CSNK2B expression increase in breast cancer and play a key role in the progression of breast cancer.^43^, ^44^ The expression of cyclins regulated by HOXC13 and knockdown of this gene resulted to cell cycle arrest and apoptosis, in the colon cancer cell lines.^45^ The roles of identified miRNAs in cancer were also determined. The miRNAs in prioritized interactions were related to CRC and other type of cancers. In this case, the miR‐30 family, miR‐504, miR‐34c, miR‐488, miR‐15b, miR‐195, miR‐497, miR‐15a, miR‐503, miR‐141, miR‐182, miR‐96, and miR‐136 were also related to invasion, proliferation, metastasis and tumor growth, or survival of several cancers including CRC.^46^, ^47^, ^48^, ^49^, ^50^, ^51^, ^52^, ^53^, ^54^, ^55^, ^56^, ^57^, ^58^, ^59^, ^60^, ^61^, ^62^, ^63^, ^64^, ^65^ However, there are few studies on the role of included prioritized miRNAs for interactions with obesity. For obesity there are some studies on mice, for instance, miR‐16‐5p decrease with high weight and a mutation in miR‐16 gene lead to increasing in body weight.^66^ The upregulation of miR‐16 was observed in calorie‐restricted mice with lower body weight.^67^ The miR‐30e‐5p and miR‐141‐3p were upregulated in high‐fat diet mice.^68^, ^69^ The miR‐30b, miR‐16, miR‐15b, and miR‐15a were downregulated in diet‐induced obesity mice.^70^ According to our results and above described documents, the miRNAs and mRNAs in obesity prioritized interactions played significant roles in CRC, this represented a strong genetic linkage on the mentioned diseases. Therefore, prioritized polymorphisms with miRNA:mRNA interactions identified in this study could be important for future investigation on the role of miRNAs and their targeted genes on CRC and obesity.

## 5 IN CONCLUSION

This was the first comprehensive systematic and bioinformatics approach for identification and prioritization of variants in miRNA binding sites of genes related to obesity or CRC as two most common complex and related diseases. The results of our study will be valuable for future association studies and functional studies to examine the role of these miRNA target site polymorphisms and genes and their association based on these identified interactions. These SNPs and interactions could be used for future studies for finding potential markers for diagnoses, prognosis, or treatment of CRC and obesity.

## CONFLICT OF INTEREST

The authors declare no conflict of interest.

## Supporting information

Supplementary Material

Supplementary Material

Supplementary Material

## Data Availability

The data that supports the findings of this study are available in the supplementary material of this article.

## References

[1] Organization WH . Obesity and overweight. https://www.who.int/news‐room/fact‐sheets/detail/obesity‐and‐overweight. 2018.

[2] Avgerinos KI , Spyrou N , Mantzoros CS , Dalamaga M . Obesity and cancer risk: emerging biological mechanisms and perspectives. Metabolism. 2019;92:121‐135. 30445141 10.1016/j.metabol.2018.11.001

[3] Gholami M , Larijani B , Zahedi Z , et al. Inflammation related miRNAs as an important player between obesity and cancers. Journal of Diabetes & Metabolic Disorders. 2019;18:675‐692. 31890692 10.1007/s40200-019-00459-2 PMC6915181

[4] Jochem C , Leitzmann M . Obesity and colorectal cancer In: Pischon T, Nimptsch K, eds. Obesity and Cancer. Recent Results in Cancer Research Vol 208 Cham: Springer; 2016:17‐41. https://doi.org/10.1007/978‐3‐319‐42542‐9_2 10.1007/978-3-319-42542-9_2 27909900

[5] Martinez‐Useros J , Garcia‐Foncillas J . Obesity and colorectal cancer: molecular features of adipose tissue. J Transl Med. 2016;14(1):21. 26801617 10.1186/s12967-016-0772-5 PMC4722674

[6] Chen C . MicroRNAs as oncogenes and tumor suppressors. N Engl J Med. 2005;353(17):1768. 16251533 10.1056/NEJMp058190

[7] Gholami M , Larijani B , Sharifi F , et al. MicroRNA‐binding site polymorphisms and risk of colorectal cancer: a systematic review and meta‐analysis. Cancer Med. 2019;8(17):7477‐7499. 31637880 10.1002/cam4.2600 PMC6885874

[8] Schneiderova M , Naccarati A , Pardini B , et al. MicroRNA‐binding site polymorphisms in genes involved in colorectal cancer etiopathogenesis and their impact on disease prognosis. Mutagenesis. 2017;32(5):533‐542. 29048575 10.1093/mutage/gex026

[9] Li J‐H , Liu S , Zhou H , Qu L‐H , Yang J‐H . starBase v2. 0: decoding miRNA‐ceRNA, miRNA‐ncRNA and protein–RNA interaction networks from large‐scale CLIP‐Seq data. Nucleic Acids Res. 2013;42(D1):D92‐D97. 24297251 10.1093/nar/gkt1248 PMC3964941

[10] Ward LD , Kellis M . HaploReg v4: systematic mining of putative causal variants, cell types, regulators and target genes for human complex traits and disease. Nucleic Acids Res. 2015;44(D1):D877‐D881. 26657631 10.1093/nar/gkv1340 PMC4702929

[11] Sabarinathan R , Tafer H , Seemann SE , Hofacker IL , Stadler PF , Gorodkin J . RNA snp: efficient detection of local RNA secondary structure changes induced by SNP s. Hum Mutat. 2013;34(4):546‐556. 23315997 10.1002/humu.22273 PMC3708107

[12] Bastami M , Nariman‐Saleh‐Fam Z , Saadatian Z , et al. The miRNA targetome of coronary artery disease is perturbed by functional polymorphisms identified and prioritized by in‐depth bioinformatics analyses exploiting genome‐wide association studies. Gene. 2016;594(1):74‐81. 27596011 10.1016/j.gene.2016.08.054

[13] Nariman‐Saleh‐Fam Z , Bastami M , Somi MH , et al. In silico dissection of miRNA targetome polymorphisms and their role in regulating miRNA‐mediated gene expression in esophageal cancer. Cell Biochem Biophys. 2016;74(4):483‐497. 27518186 10.1007/s12013-016-0754-5

[14] Krüger J , Rehmsmeier M . RNAhybrid: microRNA target prediction easy, fast and flexible. Nucleic Acids Res. 2006;34(suppl_2):W451‐W454. 16845047 10.1093/nar/gkl243 PMC1538877

[15] Ghaedi H , Bastami M , Zare‐Abdollahi D , et al. Bioinformatics prioritization of SNPs perturbing microRNA regulation of hematological malignancy‐implicated genes. Genomics. 2015;106(6):360‐366. 26520014 10.1016/j.ygeno.2015.10.004

[16] Liu C , Rennie WA , Carmack CS , et al. Effects of genetic variations on microRNA: target interactions. Nucleic Acids Res. 2014;42(15):9543‐9552. 25081214 10.1093/nar/gku675 PMC4150780

[17] Long D , Lee R , Williams P , Chan CY , Ambros V , Ding Y . Potent effect of target structure on microRNA function. Nat Struct Mol Biol. 2007;14(4):287. 17401373 10.1038/nsmb1226

[18] Marín RM , Vaníček J . Efficient use of accessibility in microRNA target prediction. Nucleic Acids Res. 2010;39(1):19‐29. 20805242 10.1093/nar/gkq768 PMC3017612

[19] The Cancer Genome Atlas; https://www.cancer.gov/

[20] Edwards SL , Beesley J , French JD , Dunning AM . Beyond GWASs: illuminating the dark road from association to function. Am J Human Genetics. 2013;93(5):779‐797. 24210251 10.1016/j.ajhg.2013.10.012 PMC3824120

[21] Ryan BM , Robles AI , Harris CC . Genetic variation in microRNA networks: the implications for cancer research. Nat Rev Cancer. 2010;10(6):389. 20495573 10.1038/nrc2867 PMC2950312

[22] Salzman DW , Weidhaas JB . SNPing cancer in the bud: microRNA and microRNA‐target site polymorphisms as diagnostic and prognostic biomarkers in cancer. Pharmacol Ther. 2013;137(1):55‐63. 22964086 10.1016/j.pharmthera.2012.08.016 PMC3546232

[23] Song C‐Q , Zhang J‐H , Shi J‐C , et al. Bioinformatic prediction of SNPs within miRNA binding sites of inflammatory genes associated with gastric cancer. Asian Pac J Cancer Prev. 2014;15:937‐943. 24568522 10.7314/apjcp.2014.15.2.937

[24] Haas U , Sczakiel G , Laufer S . MicroRNA‐mediated regulation of gene expression is affected by disease‐associated SNPs within the 3′‐UTR via altered RNA structure. RNA Biol. 2012;9(6):924‐937. 22664914 10.4161/rna.20497 PMC3495750

[25] Richardson K , Lai C‐Q , Parnell LD , Lee Y‐C , Ordovas JM . A genome‐wide survey for SNPs altering microRNA seed sites identifies functional candidates in GWAS. BMC Genom. 2011;12(1):504. 10.1186/1471-2164-12-504 PMC3207998 21995669

[26] Whiffin N , Hosking FJ , Farrington SM , et al. Identification of susceptibility loci for colorectal cancer in a genome‐wide meta‐analysis. Hum Mol Genet. 2014;23(17):4729‐4737. 24737748 10.1093/hmg/ddu177 PMC4133584

[27] Cheng TH , Thompson D , Painter J , et al. Meta‐analysis of genome‐wide association studies identifies common susceptibility polymorphisms for colorectal and endometrial cancer near SH2B3 and TSHZ1. Sci Rep. 2015;5:17369. 26621817 10.1038/srep17369 PMC4664893

[28] Xu W , Xu J , Shestopaloff K , et al. A genome wide association study on Newfoundland colorectal cancer patients’ survival outcomes. Biomarker research. 2015;3(1):6. 25866641 10.1186/s40364-015-0031-6 PMC4393623

[29] Bai Y , Wang J , Gao Z , Dai E . Identification and verification of two novel differentially expressed proteins from non‐neoplastic mucosa and colorectal carcinoma via iTRAQ combined with liquid chromatography‐mass spectrometry. Pathol Oncol Res. 2020;26:967‐976. 30927204 10.1007/s12253-019-00651-y

[30] Kong J , Shen S , Zhang Z , Wang W . Identification of hub genes and pathways in cholangiocarcinoma by coexpression analysis. Cancer Biomarkers. 2020;27(4):505‐517. 32116234 10.3233/CBM-190038 PMC12662316

[31] Ekizoglu S , Seven D , Ulutin T , Guliyev J , Buyru N . Investigation of the SLC22A23 gene in laryngeal squamous cell carcinoma. BMC Cancer. 2018;18(1):1‐7. 29703252 10.1186/s12885-018-4381-y PMC5921549

[32] Rask‐Andersen M , Karlsson T , Ek WE , Johansson Å . Genome‐wide association study of body fat distribution identifies adiposity loci and sex‐specific genetic effects. Nat Commun. 2019;10(1):1‐10. 30664634 10.1038/s41467-018-08000-4 PMC6341104

[33] Comuzzie AG , Cole SA , Laston SL , et al. Novel genetic loci identified for the pathophysiology of childhood obesity in the Hispanic population. PLoS One. 2012;7(12):e51954. 23251661 10.1371/journal.pone.0051954 PMC3522587

[34] Pulit SL , Stoneman C , Morris AP , et al. Meta‐analysis of genome‐wide association studies for body fat distribution in 694 649 individuals of European ancestry. Hum Mol Genet. 2019;28(1):166‐174. 30239722 10.1093/hmg/ddy327 PMC6298238

[35] Kichaev G , Bhatia G , Loh P‐R , et al. Leveraging polygenic functional enrichment to improve GWAS power. Am J Human Genetics. 2019;104(1):65‐75. 30595370 10.1016/j.ajhg.2018.11.008 PMC6323418

[36] Berndt SI , Gustafsson S , Mägi R , et al. Genome‐wide meta‐analysis identifies 11 new loci for anthropometric traits and provides insights into genetic architecture. Nat Genet. 2013;45(5):501‐512. 23563607 10.1038/ng.2606 PMC3973018

[37] Speliotes EK , Willer CJ , Berndt SI , et al. Association analyses of 249,796 individuals reveal 18 new loci associated with body mass index. Nat Genet. 2010;42(11):937‐948. 20935630 10.1038/ng.686 PMC3014648

[38] Cotsapas C , Speliotes EK , Hatoum IJ , et al. Common body mass index‐associated variants confer risk of extreme obesity. Hum Mol Genet. 2009;18(18):3502‐3507. 19553259 10.1093/hmg/ddp292 PMC2729668

[39] Ng MC , Graff M , Lu Y , et al. Discovery and fine‐mapping of adiposity loci using high density imputation of genome‐wide association studies in individuals of African ancestry: African Ancestry Anthropometry Genetics Consortium. PLoS Genet. 2017;13(4):e1006719. 28430825 10.1371/journal.pgen.1006719 PMC5419579

[40] Michailidou K , Lindström S , Dennis J , et al. Association analysis identifies 65 new breast cancer risk loci. Nature. 2017;551(7678):92. 29059683 10.1038/nature24284 PMC5798588

[41] Low SK , Chung S , Takahashi A , et al. Genome‐wide association study of chemotherapeutic agent‐induced severe neutropenia/leucopenia for patients in Biobank Japan. Cancer Sci. 2013;104(8):1074‐1082. 23648065 10.1111/cas.12186 PMC7657179

[42] Yi H , Wang K , Jin J‐F , et al. Elevated adenylyl cyclase 9 expression is a potential prognostic biomarker for patients with colon cancer. Med Sci Monitor. 2018;24:19. 10.12659/MSM.906002 PMC5759510 29292367

[43] Li C , Cui J , Zou L , Zhu L , Wei W . Bioinformatics analysis of the expression of HOXC13 and its role in the prognosis of breast cancer. Oncol Lett. 2020;19(1):899‐907. 31897205 10.3892/ol.2019.11140 PMC6924138

[44] Wang W , Wang X , Li C , et al. Huaier suppresses breast cancer progression via linc00339/miR‐4656/CSNK2B signaling pathway. Front Oncol. 2019;9:1195. doi: 10.3389/fonc.2019.01195 31781497 PMC6857111

[45] Kasiri S , Ansari KI , Hussain I , Bhan A , Mandal SS . Antisense oligonucleotide mediated knockdown of HOXC13 affects cell growth and induces apoptosis in tumor cells and over expression of HOXC13 induces 3D‐colony formation. RSC Adv. 2013;3(10):3260‐3269. 23495364 10.1039/C2RA22006G PMC3593253

[46] Bjørnetrø T , Redalen KR , Meltzer S , et al. An experimental strategy unveiling exosomal microRNAs 486–5p, 181a–5p and 30d–5p from hypoxic tumour cells as circulating indicators of high‐risk rectal cancer. J Extracellular Vesicles. 2019;8(1):1567219. 30728923 10.1080/20013078.2019.1567219 PMC6352936

[47] Laudato S , Patil N , Abba ML , et al. P53‐induced miR‐30e‐5p inhibits colorectal cancer invasion and metastasis by targeting ITGA6 and ITGB1. Int J Cancer. 2017;141(9):1879‐1890. 28656629 10.1002/ijc.30854

[48] Tang M , Zhou J , You L , Cui Z , Zhang H . LIN28B/IRS1 axis is targeted by miR‐30a‐5p and promotes tumor growth in colorectal cancer. J Cell Biochem. 2020;121:3720–3729. 10.1002/jcb.29529 31713927

[49] Jiang L‐H , Zhang H‐D , Tang J‐H . MiR‐30a: a novel biomarker and potential therapeutic target for cancer. J Oncol. 2018;2018:5167829. 30158978 10.1155/2018/5167829 PMC6106977

[50] Ni Q , Stevic I , Pan C , et al. Different signatures of miR‐16, miR‐30b and miR‐93 in exosomes from breast cancer and DCIS patients. Sci Rep. 2018;8(1):1‐10. 30154547 10.1038/s41598-018-31108-y PMC6113263

[51] Hojbjerg JA , Ebert EBF , Clement MS , Winther‐Larsen A , Meldgaard P , Sorensen B . Circulating miR‐30b and miR‐30c predict erlotinib response in EGFR‐mutated non‐small cell lung cancer patients. Lung Cancer. 2019;135:92‐96. 31447008 10.1016/j.lungcan.2019.07.005

[52] Rong G , Yang X , Wu H , Wu Y . miR‐150‐504‐519d inhibits the growth of human colorectal cancer cell line SW48 and downregulates c‐FLIP receptor. J Cell Biochem. 2019;120(5):7962‐7969. 10.1002/jcb.28073 30548660

[53] Li N , Mao D , Cao Y , Li H , Ren F , Li K . Downregulation of SIRT6 by miR‐34c‐5p is associated with poor prognosis and promotes colon cancer proliferation through inhibiting apoptosis via the JAK2/STAT3 signaling pathway. Int J Oncol. 2018;52(5):1515‐1527. 29512698 10.3892/ijo.2018.4304 PMC5873872

[54] Lv Y , Shi Y , Han Q , Dai G . Histone demethylase PHF8 accelerates the progression of colorectal cancer and can be regulated by miR‐488 in vitro. Molecular Med Rep. 2017;16(4):4437‐4444. 10.3892/mmr.2017.7130 PMC5647003 28765946

[55] Wang Y‐B , Shi Q , Li G , Zheng J‐H , Lin J , Qiu W . MicroRNA‐488 inhibits progression of colorectal cancer via inhibition of the mitogen‐activated protein kinase pathway by targeting claudin‐2. Am J Physiol‐Cell Physiol. 2019;316(1):C33‐C47. 30207785 10.1152/ajpcell.00047.2018

[56] Ji D , Zhan T , Li M , et al. Enhancement of sensitivity to chemo/radiation therapy by using miR‐15b against DCLK1 in colorectal cancer. Stem Cell Rep. 2018;11(6):1506‐1522. 10.1016/j.stemcr.2018.10.015 PMC6294114 30449704

[57] Sun L‐N , Zhi Z , Chen L‐Y , et al. SIRT1 suppresses colorectal cancer metastasis by transcriptional repression of miR‐15b‐5p. Cancer Lett. 2017;409:104‐115. 28923398 10.1016/j.canlet.2017.09.001

[58] Jin Y , Wang M , Hu H , Huang Q , Chen Y , Wang G . Overcoming stemness and chemoresistance in colorectal cancer through miR‐195‐5p‐modulated inhibition of notch signaling. Int J Biol Macromol. 2018;117:445‐453. 29852230 10.1016/j.ijbiomac.2018.05.151

[59] Bai J , Xu J , Zhao J , Zhang R . lncRNA SNHG1 cooperated with miR‐497/miR‐195‐5p to modify epithelial–mesenchymal transition underlying colorectal cancer exacerbation. J Cell Physiol. 2020;235(2):1453‐1468. 31276207 10.1002/jcp.29065

[60] Fesler A , Liu H , Ju J . Modified miR‐15a has therapeutic potential for improving treatment of advanced stage colorectal cancer through inhibition of BCL2, BMI1, YAP1 and DCLK1. Oncotarget. 2018;9(2):2367. 29416778 10.18632/oncotarget.23414 PMC5788646

[61] Li L , Zhang X , Yi Z , Liang X , Yin W , Li S . MiR‐503 promotes the migration and invasion of colorectal cancer cells by regulating PDCD4. J BUON. 2018;23:579‐586. 30003722

[62] Meltzer S , Bjørnetrø T , Lyckander LG , et al. Circulating exosomal miR‐141‐3p and miR‐375 in metastatic progression of rectal cancer. Transl Oncol. 2019;12(8):1038‐1044. 31146167 10.1016/j.tranon.2019.04.014 PMC6542769

[63] Jin Y , Zhang Z , Huang Y , Zhang K , Xiong B . MiR‐182‐5p inhibited proliferation and metastasis of colorectal cancer by targeting MTDH. Eur Rev Med Pharmacol Sci. 2019;23(4):1494‐1501. 30840271 10.26355/eurrev_201902_17107

[64] He PY , Yip WK , Jabar MF , Mohtarrudin N , Dusa NM , Seow HF . Effect of the miR‐96‐5p inhibitor and mimic on the migration and invasion of the SW480‐7 colorectal cancer cell line. Oncol Lett. 2019;18(2):1949‐1960. 31423265 10.3892/ol.2019.10492 PMC6607361

[65] Jin C , Wang A , Liu L , Wang G , Li G . Hsa_circ_0136666 promotes the proliferation and invasion of colorectal cancer through miR‐136/SH2B1 axis. J Cell Physiol. 2019;234(5):7247‐7256. 30370521 10.1002/jcp.27482

[66] Jia X , Lin H , Nie Q , Zhang X , Lamont SJ . A short insertion mutation disrupts genesis of miR‐16 and causes increased body weight in domesticated chicken. Sci Rep. 2016;6:36433. 27808177 10.1038/srep36433 PMC5093740

[67] Yamada K , Takizawa S , Ohgaku Y , et al. MicroRNA 16–5p is upregulated in calorie‐restricted mice and modulates inflammatory cytokines of macrophages. Gene. 2020;725:144191. 31654705 10.1016/j.gene.2019.144191

[68] Ji J , Qin Y , Ren J , et al. Mitochondria‐related miR‐141‐3p contributes to mitochondrial dysfunction in HFD‐induced obesity by inhibiting PTEN. Sci Rep. 2015;5:16262. 26548909 10.1038/srep16262 PMC4637860

[69] Miranda K , Mehrpouya‐Bahrami P , Nagarkatti PS , Nagarkatti M . Cannabinoid receptor 1 blockade attenuates obesity and adipose tissue type 1 inflammation through miR‐30e‐5p regulation of Delta‐like‐4 in macrophages and consequently downregulation of Th1 cells. Front Immunol. 2019;10:1049. 31134094 10.3389/fimmu.2019.01049 PMC6523050

[70] Hsieh C‐H , Rau C‐S , Wu S‐C , et al. Weight‐reduction through a low‐fat diet causes differential expression of circulating microRNAs in obese C57BL/6 mice. BMC Genom. 2015;16(1):699. 10.1186/s12864-015-1896-3 PMC4571067 26377847

